# Cat-Scratch Disease Presenting as Isolated Neuroretinitis (Bartonella henselae): A Case Report

**DOI:** 10.7759/cureus.95383

**Published:** 2025-10-25

**Authors:** Ke Wu, Yong Wang, Jiayuan Xing

**Affiliations:** 1 Ophthalmology, Shaanxi Eye Hospital, Xi’an People’s Hospital (Xi’an Fourth Hospital) Affiliated People’s Hospital of Northwest University, Xi'an, CHN; 2 Neuro-Ophthalmology Center, Shaanxi Eye Hospital, Xi’an People’s Hospital (Xi’an Fourth Hospital) Affiliated People’s Hospital of Northwest University, Xi‘an, CHN

**Keywords:** bartonella henselae, cat-scratch disease, macular stellate exudate, neuroretinitis, vision impairment

## Abstract

Cat-scratch disease (CSD), a rare infectious disorder typically presenting with systemic manifestations such as fever and lymphadenopathy, uncommonly involves ocular structures, with neuroretinitis being one of its more frequent ocular manifestations. We report an unusual case of CSD presenting as isolated neuroretinitis in the absence of constitutional symptoms. The patient is an 18-year-old female who presented with decreased vision in her left eye and the appearance of shadows. She reported no systemic symptoms such as fever or swollen lymph nodes. Fundus examination revealed optic disc edema, retinal edema in the posterior pole, and macular star exudates. Diagnosis was confirmed by a history of cat exposure and positive serology for *Bartonella henselae* antibodies. Treatment with oral doxycycline, combined with azithromycin and low-dose glucocorticoids, resulted in significant visual improvement. This case highlights that CSD should be considered in cases of unexplained neuroretinitis, and a careful history regarding cat contact is essential to avoid misdiagnosis.

## Introduction

Cat-scratch disease (CSD), also known as cat-scratch fever, is a self-limited, systemic infectious disease caused by *Bartonella henselae* (*B. henselae*), a small, fastidious, gram-negative intracellular bacillus. It typically occurs in immunocompetent patients younger than 20 years of age. Humans are infected through cat scratches, licks, or bites [1,2]. Systemic manifestations of CSD typically include fever, tender lymphadenopathy, and cutaneous lesions, sometimes accompanied by headache, arthralgia, or anorexia. However, ocular complications arise in merely 5-10% of patients. These encompass a range of conditions, such as uveitis, neuroretinitis, retinal vessel occlusions, retinal detachment, and Parinaud's oculoglandular syndrome (POGS), of which neuroretinitis is one of the common manifestations [3,4]. All of the known reported patients with ocular manifestations of CSD have been combined with fever or other systemic manifestations [5-8]. Patients presenting with isolated optic retinitis have not been reported.

## Case presentation

**Figure 1:**
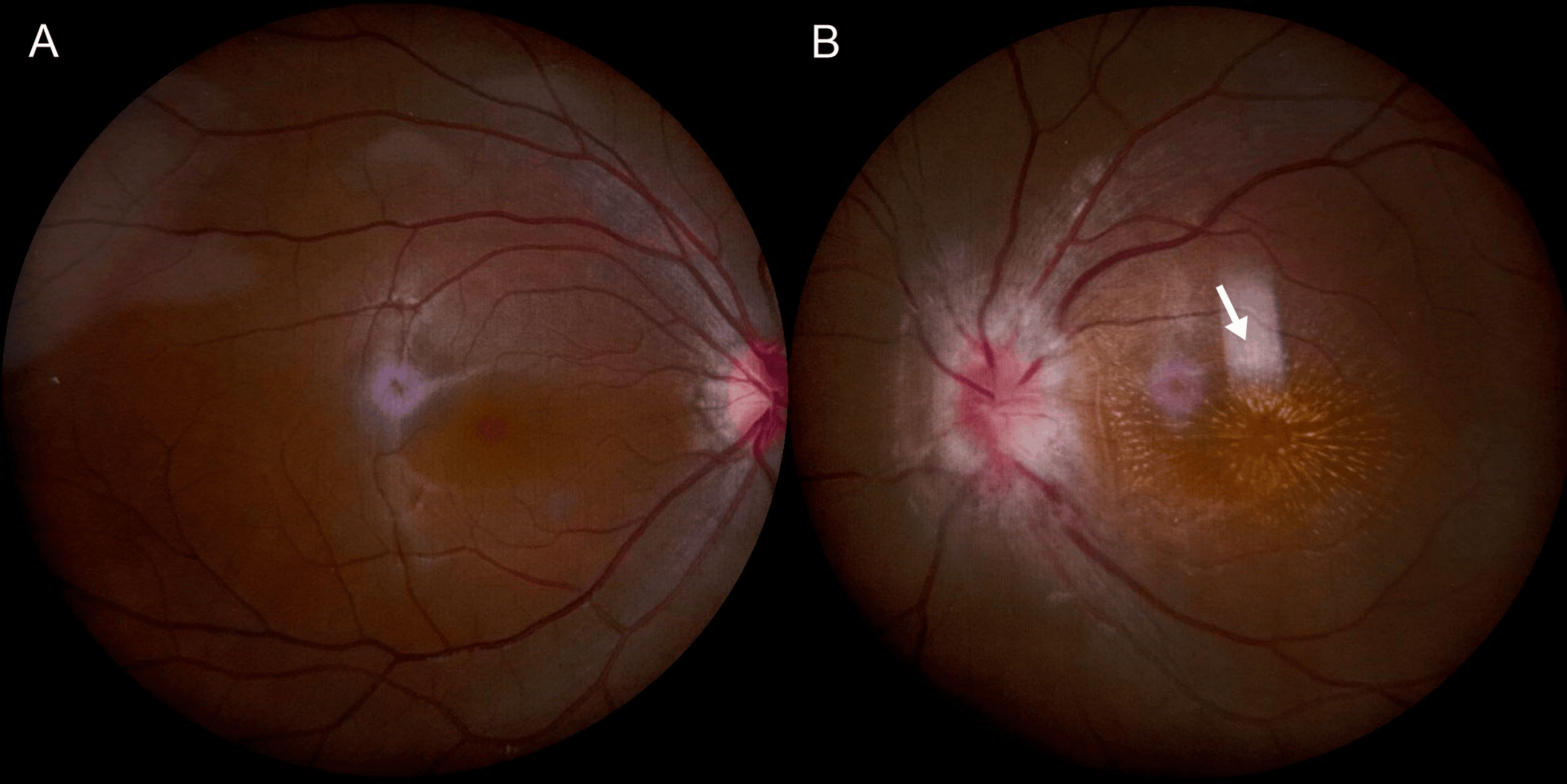
Fundus photographs of both eyes are presented. The right eye (Panel A) is the healthy eye, while the left eye (Panel B) is the affected eye, showing optic disc congestion and edema with blurred margins, as well as stellate exudates in the macular region (white arrow).

The patient's chief complaint was decreased left eye vision accompanied by a central shadow, lasting for one week in August 2025. She denied systemic symptoms, such as fever or headache, and reported no localized signs, including lymph node enlargement, tenderness, cutaneous nodules, or pruritus. At the time of admission, the best-corrected visual acuity (BCVA) in the left eye was 0.1, the pupil diameter was 4 mm, and the relative afferent pupillary defect (RAPD) was positive. The fundus showed a congested and edematous optic disc with unclear borders and stellate exudates in the macula (fundus photography can be seen in Figure 1).

**Figure 2:**
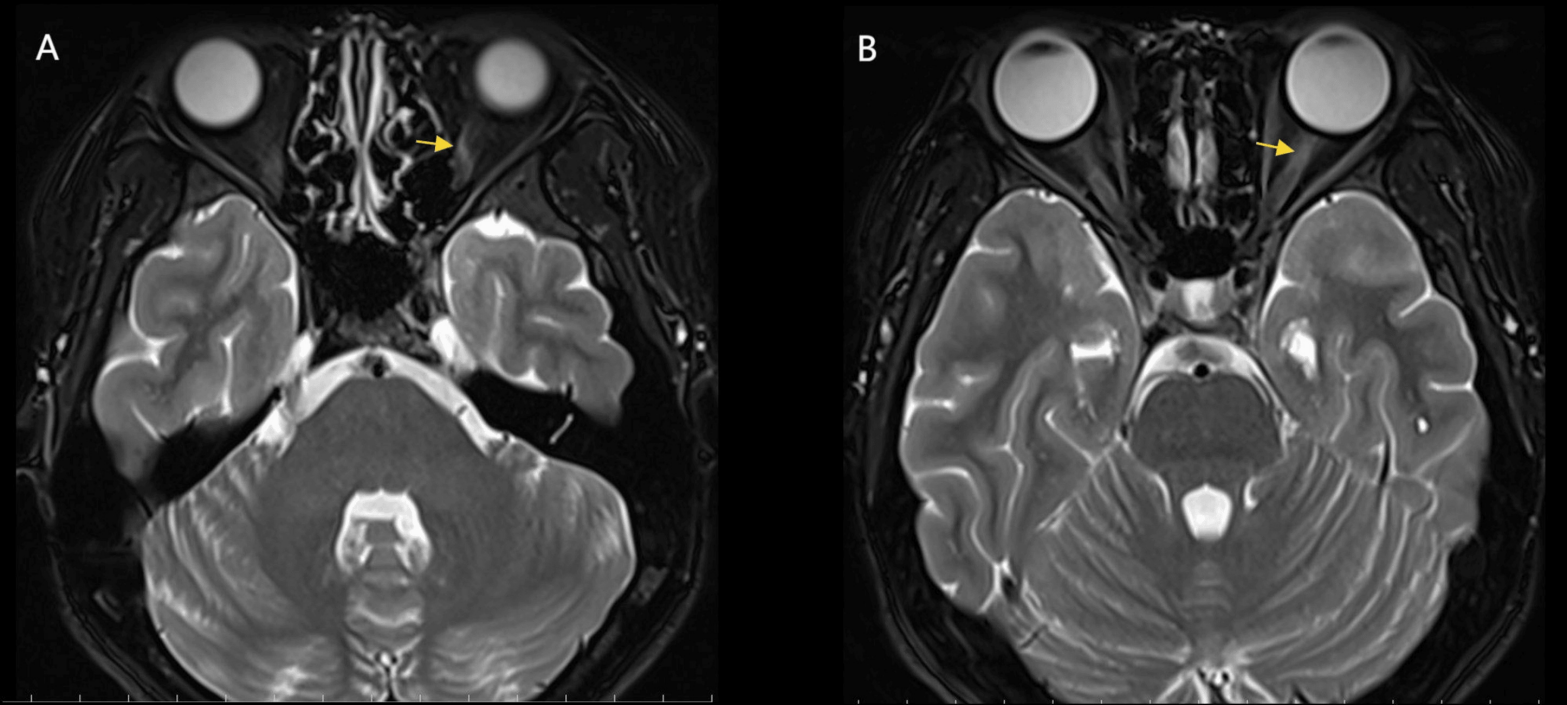
Orbital MRI demonstrated a hyperintense signal on T2-weighted imaging in the anterior intraorbital segment of the left optic nerve (yellow arrows in panels A and B). MRI, magnetic resonance imaging.

To elucidate the cause of the disease, the patient underwent a systematic investigation. The workup included comprehensive laboratory tests, imaging of the head and orbits, and a detailed ophthalmological assessment. Blood tests were no abnormalities in routine blood tests, calcitoninogen, liver function, renal function, coagulation, infectious disease series (including hepatitis B, hepatitis C, syphilis, and AIDS), cytokine tests, and TORCH tests (Including *Toxoplasma gondii*, rubella virus, cytomegalovirus, herpes simplex virus types I and II), while the blood sedimentation was elevated by 28 mm/h (the normal range is less than 20 mm/h), and C-reactive protein was elevated by 14.2 mg/L (the normal range is less than 6 mg/L). Brain magnetic resonance imaging (MRI) showed no significant abnormality, while orbital MRI showed a slightly high signal on T2-weighted imaging (T2WI) in the anterior segment of the intraorbital segment of the optic nerve in the left eye (Figure 2).

**Figure 3:**
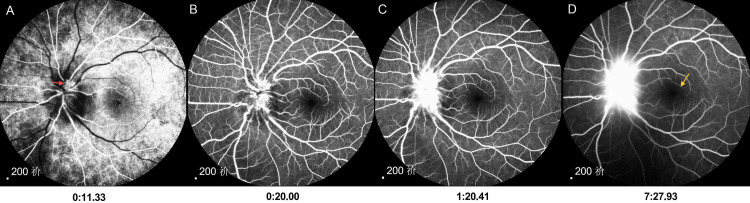
FFA shows early optic disc capillary filling and dilatation in the left eye (red arrow in panel A), fluorescein leakage from the optic disc was gradually evident in the middle and late stages (panels B-D), and no significant leakage was seen in the macula throughout (yellow arrow in panel D). FFA, fundus fluorescein angiography.

**Figure 4:**
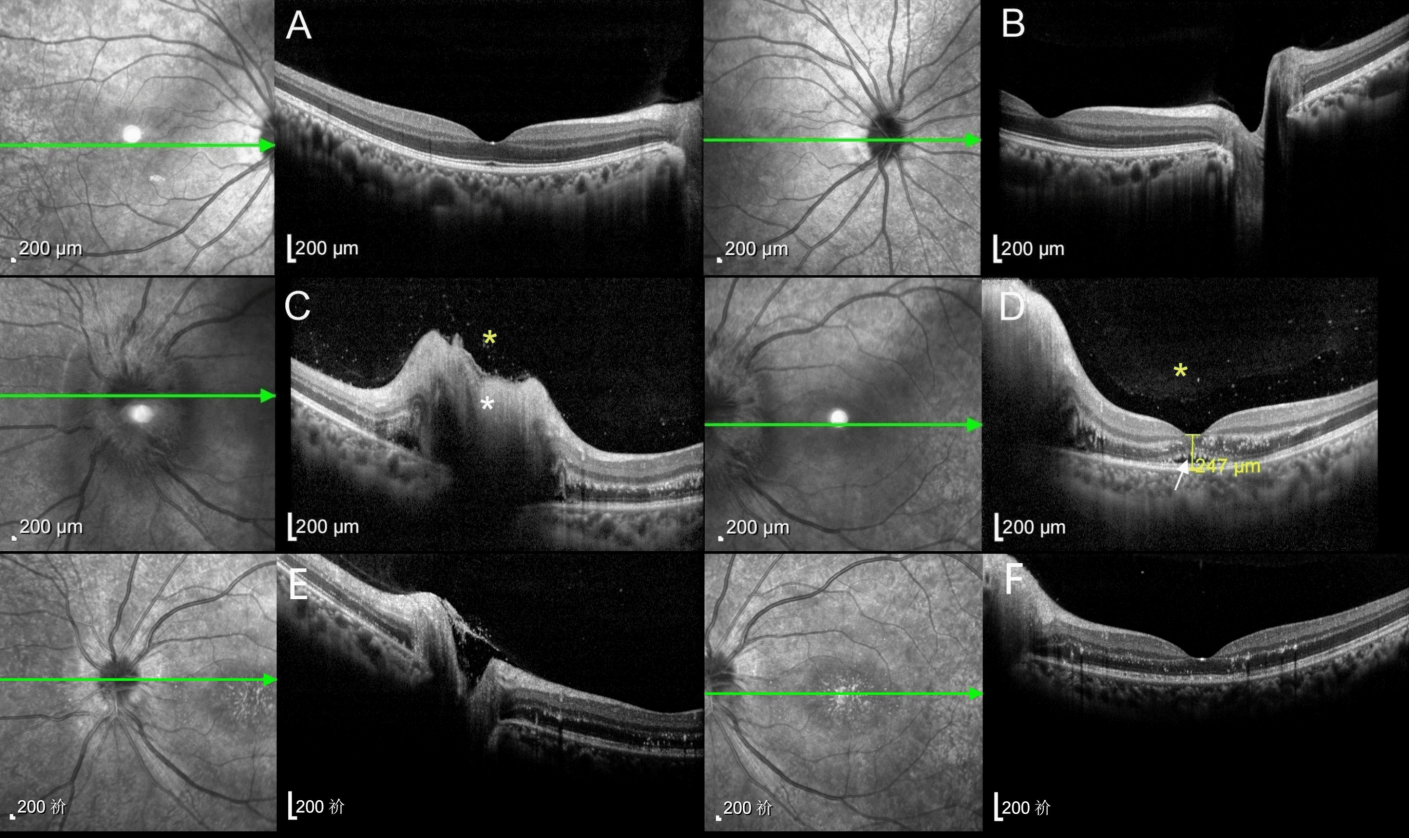
OCT images of both eyes are presented. The right eye (Panels A-B) is normal, while the left eye (Panels C-D) shows abnormal findings: an uneven vitreous signal with scattered punctate hyperreflective foci (yellow asterisks in C-D), thickening of the neuroepithelial layer with subretinal fluid accumulation in the optic disc region (white asterisks in C), and retinal detachment in the macular area with granular hyperreflective deposits in the outer retinal layers (white arrow in D). After two weeks of treatment (panels E-F), the height of optic disc and macular edema in the patient's left eye decreased compared to the previous measurement, and the retinal layers became more clearly defined than before. OCT, optical coherence tomography.

Fundus fluorescein angiography (FFA, Figure 3) showed that there was early capillary dilatation of the optic disc in the left eye, with significant fluorescein leakage in the mid to late stages, and no significant fluorescein leakage in the macula throughout. Optical coherence tomography (OCT, Figure 4) showed an uneven vitreous signal and a visible scattered punctate high signal in the left eye, thickening of the neuroepithelial layer in the region of the optic disc, accumulation of subretinal fluid, retinal detachment in the macular region, and granular high-signal deposits in the outer layers.

Combining all of the above, we initially diagnosed “neuroretinitis” and gave intravenous steroid therapy with methylprednisolone, accompanied by improvement of circulation, nutritional nerve, and other treatments; after two days of medication, the patient complained of a further decline in vision in the left eye, and the BCVA was 0.02 only. We were very puzzled by this, and after further inquiries about the patient's medical history, the patient admitted to having adopted a stray cat half a year earlier and denied any history of cat scratches, but had been licked by the cat. Therefore, we highly suspected the possibility of CSD and conducted a serologic *B. henselae* antibody test, which showed a positive result with a titer of 1:256, indicating that the patient had recently been infected with Bartonella.

**Figure 5:**
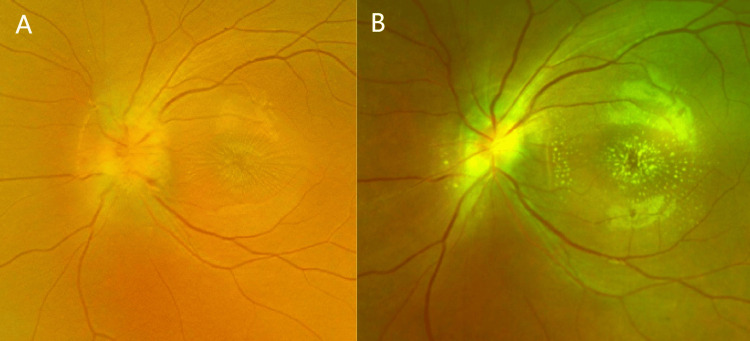
Fundus photographs at admission (Panel A) and after two weeks of treatment (Panel B) are shown. Compared to admission, post-treatment optic disc edema has significantly resolved, with its margins becoming markedly clearer. Stellate macular exudates persist, but their borders and coloration are now more distinct than they were previously.

Finally, we gave the patient doxycycline supplemented with azithromycin and a small dose of glucocorticoids, and after two days, the patient's visual acuity began to improve. After two weeks of treatment, we reviewed the patient's fundus photographs (Figure 5) and OCT images (Figure 4, panels E, F). Compared to the admission findings, the fundus photographs showed significant resolution of optic disc edema with markedly improved border clarity. The stellate macular exudates persisted, but their borders and coloration were more distinct than previously observed. The OCT comparison also demonstrates improvement in the patient's condition. The height of both the optic disc and macular edema has decreased compared to previous measurements, and the retinal layer structures appear clearer than before. The patient's uncorrected visual acuity in the left eye recovered to 0.1, with BCVA reaching 0.3, and she reported that the darkness in front of her eye had noticeably faded.

## Discussion

CSD is a self-limiting infectious disease primarily caused by *B. henselae* infection. Ocular manifestations of CSD encompass a spectrum of conditions, including uveitis, neuroretinitis, vasculitis, retinal vessel occlusions, retinal detachment, and POGS [9]. Neuroretinitis is defined as inflammation of the optic nerve and peripheral retina of the optic disc, and is usually recognized clinically by swelling of the optic disc and deposition of lipid-rich inflammatory exudate from the retina in a radial pattern, resulting in a macular stellate exudate appearance [3,7,10], but there is no fluorescein leakage in the macula on FFA examination. Cells and flashes in the anterior chamber are sometimes visible, and mild vitreous is common. Vision loss is the most common ocular symptom, with patients presenting with visual acuity ranging from light to 1.0, usually with RAPD, visual field defects, and color vision deficits.

The exact pathogenesis of CSD-associated neuroretinitis remains to be elucidated. Optic nerve involvement may be caused by optic nerve or intraocular Bartonella infection, an immune response to bacterial infection, or a combination of infectious and noninfectious mechanisms [11]. Inflammation of the optic disc leads to fluid leakage into the peripapillary retina, resulting in the formation of a plasma retinal detachment, followed by macular exudates in a partial or complete stellate pattern around the central pits. The diagnoses of the disease include young patients, a history of cat contact, typical neuroretinitis, systemic symptoms, and positive serologic tests. The indirect fluorescent antibody (IFA) test is the most reliable method with a specificity of 95%.

Neuroretinitis caused by CSD is characterized by obvious edema of the optic disc, retinal edema in the posterior pole with stellate exudation in the macula, and fluorescent leakage from the optic disc but not from the macula on FFA [3]. The patient in this case is consistent with this typical presentation. In this report, the patient only presented with monocular neuroretinitis but did not have systemic manifestations such as fever, rash, or lymph node enlargement, which made it very easy to miss the diagnosis at the initial diagnosis. This case suggests to ophthalmologists that this disease should be suspected when encountering patients with clinical manifestations of isolated neuroretinitis and that they need to follow up with a history of cat ownership or exposure, and if necessary, perform serologic testing to ensure accurate diagnosis and effective treatment of the disease.

There is no consensus on the treatment of CSD. In immunocompetent patients, CSD usually presents with a self-limiting course. However, immunocompromised patients with severe ocular symptoms or systemic complications require treatment. Antibiotic choices may include doxycycline, azithromycin, erythromycin, ciprofloxacin, methotrexate-sulfamethoxazole, gentamicin, or rifampin [1,3]. Recent data also suggest that the use of oral corticosteroids in combination with antibiotics may further improve visual prognosis in patients with CSD neuroretinitis. Since doxycycline has good intraocular penetration and our patient presented with only ocular symptoms, we preferred doxycycline orally, supplemented with azithromycin and small doses of glucocorticoids, and achieved good clinical results [12,13].

Our case study also has its limitations. During the initial medical history collection for this unexplained optic neuroretinitis, we failed to inquire about the cat's exposure history. As a result, the patient's vision continued to deteriorate two days after the initial treatment. This serves as a reminder to clinicians that when encountering unusual fundus changes, a thorough medical history must be obtained to establish a definitive diagnosis and prevent treatment delays.

## Conclusions

We report a rare case of CSD in a patient who presented without typical symptoms such as fever or lymphadenopathy, exhibiting only decreased ocular vision and a dark shadow in the anterior segment of the left eye. The diagnosis was ultimately confirmed through a history of cat exposure and serological testing. This case suggests to ophthalmologists that this disease should be suspected when encountering patients with clinical manifestations of isolated neuroretinitis and that they need to follow up with a history of cat ownership or exposure, and, if necessary, perform serologic testing to ensure accurate diagnosis and effective treatment of the disease.
